# Chalcopyrite CuFeS_2_: Solid-State Synthesis and Thermoelectric Properties

**DOI:** 10.3390/ma17225497

**Published:** 2024-11-11

**Authors:** Jin-Sol Kim, Il-Ho Kim

**Affiliations:** Department of Materials Science and Engineering, College of Engineering, Korea National University of Transportation, Chungju 27469, Republic of Korea; jinsol0315@a.ut.ac.kr

**Keywords:** thermoelectric, chalcopyrite, solid-state synthesis

## Abstract

The optimal conditions for synthesizing a pure chalcopyrite CuFeS_2_ phase were thoroughly investigated through the combination of mechanical alloying (MA) and hot pressing (HP) processes. The MA process was performed at a rotational speed of 350 rpm for durations ranging from 6 to 24 h under an Ar atmosphere, ensuring proper mixing and alloying of the starting materials. Afterward, MA-synthesized chalcopyrite powder was subjected to HP at temperatures between 723 K and 823 K under a pressure of 70 MPa for 2 h in a vacuum. This approach aimed to achieve phase consolidation and densification. A thermal analysis via differential scanning calorimetry (DSC) revealed distinct endothermic peaks at the range of 740–749 K and 1169–1170 K, corresponding to the synthesis of the chalcopyrite phase and its melting point, respectively. An X-ray diffraction (XRD) analysis confirmed the successful synthesis of the tetragonal chalcopyrite phase across all samples. However, a minor secondary phase, identified as Cu_1.1_Fe_1.1_S_2_ (talnakhite), was observed in the sample hot-pressed at the highest temperature of 823 K. This secondary phase could result from slight compositional deviations or local phase transformations at elevated temperatures. The thermoelectric properties of the CuFeS_2_ samples were evaluated as a function of the HP temperatures. As the HP temperature increased, the electrical conductivity exhibited a corresponding rise, likely due to enhanced densification and reduced grain boundary resistance. However, this increase in electrical conductivity was accompanied by a decrease in both the Seebeck coefficient and thermal conductivity. The reduction in the Seebeck coefficient could be attributed to the higher carrier concentration resulting from improved electrical conductivity, while the decrease in thermal conductivity was likely due to reduced phonon scattering facilitated by the grain boundaries. Among the samples, the one that was hot-pressed at 773 K displayed the most favorable thermoelectric performance. It achieved the highest power factor of 0.81 mWm^−1^K^−1^ at 523 K, indicating a good balance between the Seebeck coefficient and electrical conductivity. Additionally, this sample achieved a maximum figure-of-merit (ZT) of 0.32 at 723 K, a notable value for chalcopyrite-based thermoelectric materials, indicating its potential for mid-range temperature applications.

## 1. Introduction

AMX_2_ chalcogenide compounds (A = Cu and Ag; M = Al, Ga, In, and Fe; X = S, Se, and Te) are versatile materials with applications in photoelectronic conversion and nonlinear optical properties [1,2,3]. These properties can be fine-tuned through ion substitution, particularly with M or X ions. Recent investigations have extended the utility of AMX_2_ compounds to thermoelectric applications due to their favorable structural and electronic characteristics [4,5,6]. Among these, chalcopyrite (CuFeS_2_), with a band gap of approximately 0.53 eV, stands out as a promising *n*-type thermoelectric material [7]. Its negative Seebeck coefficient allows for carrier concentration adjustment through doping, offering potential for optimizing thermoelectric performance [5,6].

Chalcopyrite is not only abundant and eco-friendly but also possesses a low toxicity profile, making it an ideal candidate for sustainable material applications. It crystallizes in the tetragonal space group I $\bar{4}$2d (a = 0.5289 nm, c = 1.0423 nm, c/a = 1.971) and the constituent atoms occupy the following Wyckoff positions: Cu at 4a (0, 0, 0), Fe at 4b (0, 0, 1/2), and S at 8d (1/4, 0, 1/2) [8], which is associated with its thermoelectric and semiconductor properties [9,10,11]. Given the increasing focus on environmentally friendly thermoelectric materials for large-scale production, the sulfide family holds great promise [12]. Several copper sulfides, such as Cu_2_S [13], Cu_5_FeS_4_ [14], and Cu_2_SnS_3_ [15], have been explored for their thermoelectric properties due to their *p*-type behavior, typically driven by copper vacancies ($V_{Cu}^{-}$), which promote hole conductivity [16].

For the realization of full thermoelectric devices, both *p*-type and *n*-type materials are required. While many copper sulfides exhibit *p*-type characteristics, chalcopyrite (CuFeS_2_) is an intrinsic *n*-type semiconductor. The *n*-type behavior arises from sulfur vacancies ($V_{S}^{‥}$), which introduce free electrons as charge carriers [17,18]. Furthermore, CuFeS_2_ exhibits antiferromagnetic behavior due to the magnetic moments of Fe^3+^, with a Néel temperature of 823 K, making it interesting not only for thermoelectricity but also for magnetic applications [19]. Some studies suggest that hybridization between the d-orbitals of Fe and the sp-orbitals of S leads to the formation of additional bands near the valence band, potentially enhancing carrier mobility [20].

In this study, CuFeS_2_ was synthesized using mechanical alloying (MA), a technique known for its ability to prevent chalcogen loss, which can occur during high-temperature melting processes. Despite the possibility of generating undesired crystalline defects and amorphous particles, MA is particularly advantageous for synthesizing chalcogenide compounds as it is energy-efficient and time-saving. The MA process was followed by hot pressing (HP) to consolidate the powder into dense specimens. This approach allowed for a detailed investigation into the effects of MA and HP processing conditions on phase transitions and the resulting thermoelectric properties of CuFeS_2_. By optimizing these processes, it is possible to fine-tune the microstructure and thermoelectric behavior of chalcopyrite, paving the way for its use in practical thermoelectric devices.

## 2. Experimental Procedure

To synthesize CuFeS_2_, Cu (purity 99.9%, particle size < 45 μm, Kojundo, Japan), Fe (purity 99.9%, particle size < 53 μm, Kojundo, Japan), and S (purity 99.99%, particle size < 75 μm, Kojundo, Japan) powders were weighed according to the stoichiometric ratio, with a total weight of 20 g. A planetary ball mill (Pulverisette5, Fritsch, Pittsboro, NC, USA) consisting of stainless steel balls and vessels was utilized. The mixed powders were placed in a hardened steel jar along with 400 g of steel balls, and MA was carried out at a rotational speed of 350 rpm under an Ar atmosphere for durations ranging from 6 to 24 h. Thermal analysis of the synthesized MA powder was performed using thermogravimetric and differential scanning calorimetry (TG–DSC; TGA/DSC1, Mettler Toledo, Columbus, OH, USA) at temperatures ranging from 300 to 1273 K with a heating rate of 5 K min^−1^. The HP process was applied to the MA powder, which was placed in a graphite mold with an inner diameter of 10 mm. HP was conducted under vacuum, applying a pressure of 70 MPa, at temperatures ranging from 723 to 823 K for 2 h to consolidate the powder. The specimen names prepared in this study, along with the MA and HP processing variables, are provided in Table 1.

Phase identification and lattice parameter analysis were performed on both MA powders and HP specimens using X-ray diffraction (XRD; D8-Advance, Bruker, Billerica, MA, USA) with Cu Kα radiation. The results were further analyzed using Rietveld refinement software (TOPAS ver. 4.1). Microstructural observations of the HP samples were carried out using scanning electron microscopy (SEM; Prisma E, Thermo Fisher Scientific, Waltham, MA, USA), and their compositions were assessed through energy-dispersive spectroscopy (EDS; Quantax 200, Bruker, Billerica, MA, USA). The sintered body was cut into a rectangular parallelepiped with dimensions of 3 × 3 × 9 mm to measure the Seebeck coefficient and electrical conductivity, and then it was cut into disks with dimensions of 10 mm (diameter) × 1 mm (thickness) for thermal conductivity. The Seebeck coefficient (α) and electrical conductivity (σ) were measured using the DC four-probe method in a helium atmosphere with a ZEM-3 (Advance Riko, Yokohama, Japan) instrument. The thermal diffusivity (D) of the specimens was determined using the laser flash method (TC-9000H, Advance Riko). Thermal conductivity (κ) was then calculated based on the equation, κ = D∙c_p_∙d, where c_p_ is the specific heat and d is the density of the material. The specific heat of the sample was 0.52 Jg^−1^K^−1^ [16]. Subsequently, the power factor (PF = α^2^σ) and the dimensionless figure of merit (ZT = PF∙κ^–1^∙T) were evaluated over the temperature (T) range of 323–723 K to assess the performance of the synthesized CuFeS_2_ material.

## 3. Results and Discussion

**Figure 1:**
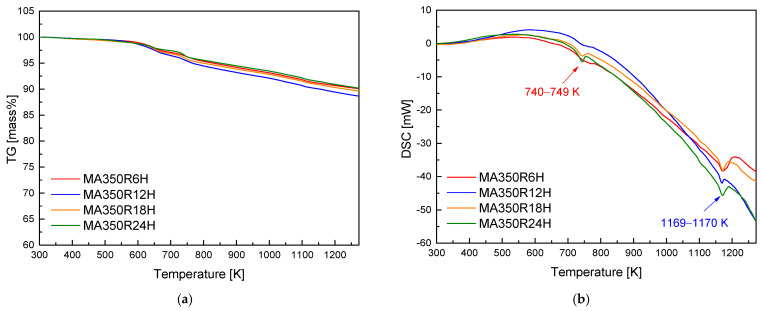
Thermal analysis plots via (**a**) TG and (**b**) DSC of CuFeS_2_ powders synthesized by MA.

Figure 1 presents the TG–DSC curves and thermal analysis of CuFeS_2_ powders as a function of the MA time. The thermal behavior of the powders shows two distinct endothermic peaks, one between 740 and 749 K and another between 1169 and 1170 K. These peaks signify the decomposition and melting of CuFeS_2_ due to sulfur volatilization at higher temperatures. The endothermic peak at 740–749 K corresponds to the synthesis of CuFeS_2_ from residual elements during the heating process, while the peak at 1169–1170 K indicates the melting point of CuFeS_2_. Barton [21] and Frenzel et al. [22] studied the phase diagram of the Cu-Fe-S system; they found many kinds of phases (FeS_2_, Cu_5_FeS_4_, and CuFeS_2_) and discovered that CuFeS_2_ decomposes at 830 K.

Previous studies, such as that by Xie et al. [5], reported that CuS formed at around 393 K and reacted with S and Fe to produce CuFeS_2_ at approximately 758 K, with oxidation–reduction reactions occurring above 781 K. Similarly, Li et al. [11] suggested that during the early stages of MA, the energy from ball collisions was insufficient for Fe to react immediately with Cu and S, resulting in the initial formation of CuS, which then transformed into CuFeS_2_ as the milling time and energy increased. However, in the current study, the direct synthesis of CuFeS_2_ was achieved without the formation of intermediate phases like CuS. This suggests that sufficient energy was provided during MA for Cu, Fe, and S to react directly, resulting in the immediate formation of CuFeS_2_. Li et al. [21] also observed that the vaporization and decomposition of CuFeS_2_ began at around 800 K in the DSC analysis. Ge et al. [16] reported that in their TG analysis, no significant weight loss occurred for CuFeS_2_ up to 760 K, but above this temperature, sulfur volatilization began. However, given the high specific surface area of the fine powders in this study, it is anticipated that volatilization will occur and intensify with increasing temperature. Ge et al. [16] performed thermal analyses on sintered compacts rather than powders. Their findings indicate that the weight loss due to volatilization was lower at elevated temperatures compared to our powder samples, and the phase decomposition temperature was also higher.

**Figure 2:**
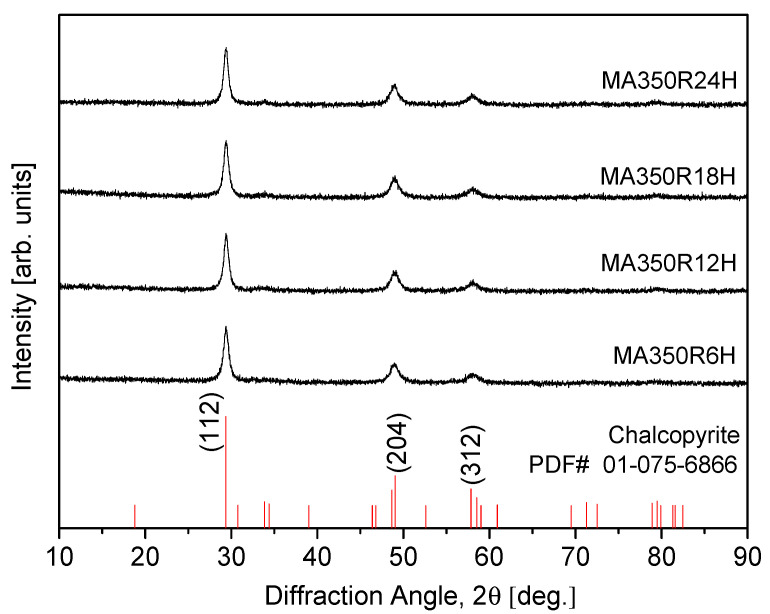
XRD patterns of CuFeS_2_ powders synthesized using MA.

Figure 2 shows the XRD patterns and phase analysis of the synthesized MA powders processed at varying milling times. It was observed that as the MA time increased, the average particle size decreased. Using the TOPAS program to measure the Lorentzian crystallite size, it slightly decreased from 14.5 nm (MA350R6H) to 12.8 nm (MA350R24H) as shown in Table 2. However, compared to the particle sizes of the raw elemental powders, the sizes of the MA-synthesized powders were significantly reduced. Notably, for the sample milled for 6 h (MA350R6H), the chalcopyrite phase (CuFeS_2_; PDF# 01-075-6866) was successfully synthesized, with no detectable secondary phases. This observation suggests that a 6 h milling duration is adequate for the complete formation of the chalcopyrite structure, indicating an efficient alloying process. Further extending the milling time to 24 h (MA350R24H) did not yield any additional phase transformations.

The absence of new phases or structural alterations under prolonged milling conditions demonstrates the stability of the CuFeS_2_ phase. This consistency in phase composition across different milling times suggests that additional milling beyond 6 h does not significantly impact the structural integrity or lead to the decomposition of the chalcopyrite phase. The findings underscore the importance of optimizing milling parameters to achieve the desired phase without unnecessary energy expenditure. However, in this study, the chalcopyrite phase was synthesized after 6 h of MA, while the powders used for the HP process underwent 12 h of MA. This extended processing time was implemented to account for potential undetected residual elements that may not have fully reacted, considering the limitations of the XRD analysis. In conjunction with the thermal analysis results obtained from DSC and the XRD phase analysis, the optimal conditions for MA were determined to be 350 rpm for 12 h. These optimized conditions were subsequently employed as a baseline for further investigations into the effects of subsequent HP conditions on both the phase evolution and thermoelectric properties of the synthesized CuFeS_2_ material.

**Figure 3:**
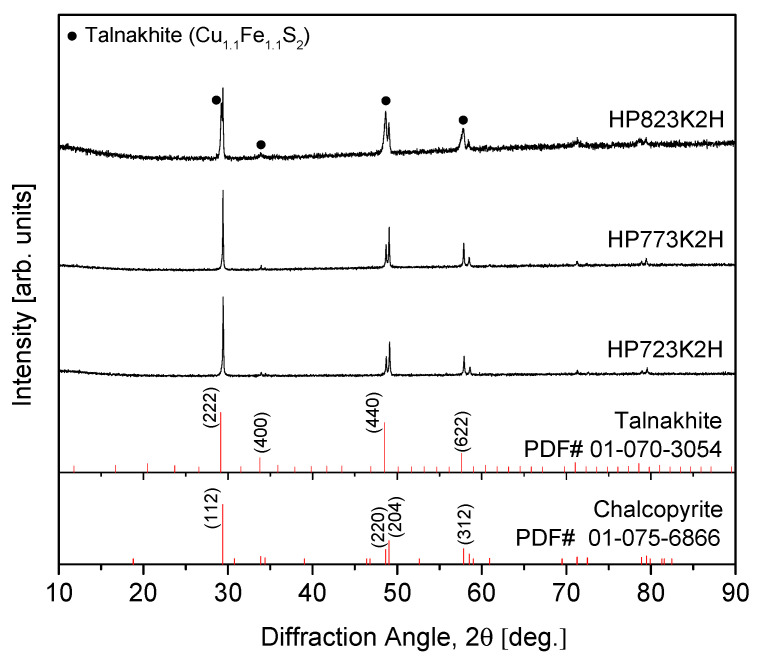
XRD patterns of CuFeS_2_ compacts prepared using the MA–HP process.

Figure 3 depicts the XRD patterns of the sintered specimens as a function of the HP temperature. The specimens sintered at 723 K (HP723K2H) and 773 K (HP773K2H) exhibited a single-phase chalcopyrite structure (CuFeS_2_), which confirms the successful preservation of the desired phase during the sintering process at these temperatures. This is significant, as it indicates that appropriate control of HP conditions can effectively maintain the integrity of the chalcopyrite phase. In contrast, when the HP temperature was elevated to 823 K (HP823K2H), a secondary phase, talnakhite (Cu_1.1_Fe_1.1_S_2_; PDF# 01-070-3054), was detected. The emergence of this secondary phase implies that the increased sintering temperature instigated sulfur volatilization, which consequently facilitated the transformation of the chalcopyrite phase into the talnakhite structure. This observation aligns with the findings of Li et al. [23], who reported that a decrease in the sulfur content is a driving force behind the transformation of CuFeS_2_ from its tetragonal chalcopyrite structure to a cubic phase. The volatilization of sulfur at elevated HP temperatures was pivotal in triggering this structural change, leading to a partial transformation from the tetragonal chalcopyrite phase to the cubic talnakhite phase. Such transformations could potentially adversely affect the thermoelectric properties of the material, as the unique electronic and thermal transport characteristics of chalcopyrite may not be retained in the talnakhite phase. Therefore, it can be concluded that to maintain the integrity of the chalcopyrite phase, the optimal HP temperature should not exceed 773 K.

The lattice parameters of CuFeS_2_ synthesized via MA–HP are summarized in Table 2 as follows: a = 0.5289–0.5292 nm, c = 1.0419–1.0438 nm, and tetragonality (c/a ratio) ranging from 1.9697 to 1.9724. These values exhibit a close agreement with the standard diffraction data for chalcopyrite (PDF# 01-075-6866), which specifies lattice parameters of a = 0.5289 nm, c = 1.0423 nm, and a tetragonality of 1.97069. This similarity indicates that the synthesized CuFeS_2_ successfully retains the characteristic tetragonal structure of chalcopyrite. Li et al. [11] emphasized that CuFeS_2_ can exist in both tetragonal (space group I $\bar{4}$2d) and cubic (space group Pn3m) forms, with the lattice parameter a = 0.5289 nm being applicable to both structures. Ge et al. [16] also reported analogous lattice constants for CuFeS_2_, specifically a = 0.5289 nm and c = 1.0420 nm, resulting in a tetragonality of 1.9701. The close correspondence between these findings and the lattice parameters observed in this study reinforces the successful synthesis of the tetragonal CuFeS_2_ phase. The slight variations in the lattice parameters reported across different studies can be attributed to differences in synthesis methods, starting materials, and processing conditions. Such variations are common in the characterization of complex materials like CuFeS_2_, reflecting the nuances of how specific parameters can influence crystal growth and structural stability.

**Figure 4:**
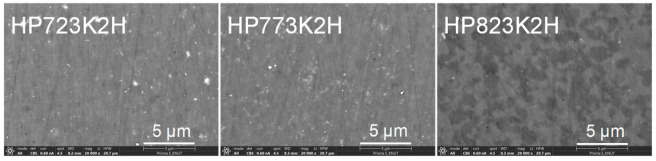
SEM images of CuFeS_2_ synthesized using the MA–HP process.

Figure 4 presents the microstructures of the CuFeS_2_ specimens sintered at different HP temperatures. The sintered samples exhibited high relative densities, ranging from 96.9% to 99.2%, with no visible pores or cracks, as summarized in Table 2. The high relative densities indicate effective densification, which is directly correlated with the sintering temperature. Notably, the theoretical density of pure CuFeS_2_ is reported as 4.19 gcm^−3^ [24]. As the HP temperature increased, the densification improved, suggesting a more compact microstructure. In particular, the samples sintered at 723 K (HP723K2H) and 773 K (HP773K2H) exhibited only the chalcopyrite phase (CuFeS_2_), consistent with a fully densified and homogenous structure. However, a different microstructure was observed for the sample sintered at 823 K (HP823K2H), where backscattered electron imaging revealed distinct dark and bright regions. These microstructural differences indicate the formation of multiple phases at higher sintering temperatures, likely due to elemental redistribution or decomposition.

**Figure 5:**
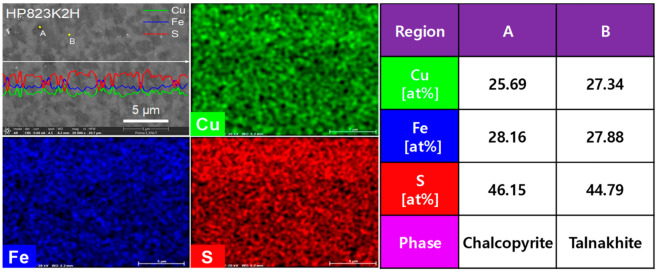
EDS elemental analysis of HP823K2H specimen.

The EDS analysis, shown in Figure 5, confirmed phase segregation in the HP823K2H sample. The dark regions (A) were identified as chalcopyrite (CuFeS_2_), while the bright regions (B) corresponded to talnakhite (Cu_1.1_Fe_1.1_S_2_), characterized by a lower sulfur content. This phase separation can be explained by sulfur volatilization at elevated temperatures, which led to the transformation of the chalcopyrite phase into talnakhite. The formation of talnakhite was further corroborated by an XRD analysis (Figure 3), where the presence of the Cu_1.1_Fe_1.1_S_2_ phase was detected in the HP823K2H sample. The observation of phase transformation at higher temperatures is consistent with previous reports. Li et al. [23] described that sulfur volatilization could induce a phase transition in CuFeS_2_, leading to the formation of cubic phases such as talnakhite, particularly when the sulfur content decreases. This study aligns with these findings, as sulfur loss during high-temperature HP resulted in the partial transformation of the tetragonal chalcopyrite phase to the cubic talnakhite phase. Furthermore, Li et al. [11] discussed the influence of sulfur vacancies on structural stability, showing that when sulfur deficiencies become significant, phase transformation from a tetragonal structure to a cubic structure can occur. The results of this study provide additional evidence for this mechanism, as sulfur volatilization likely triggered the observed transformation in the HP823K2H sample.

**Figure 6:**
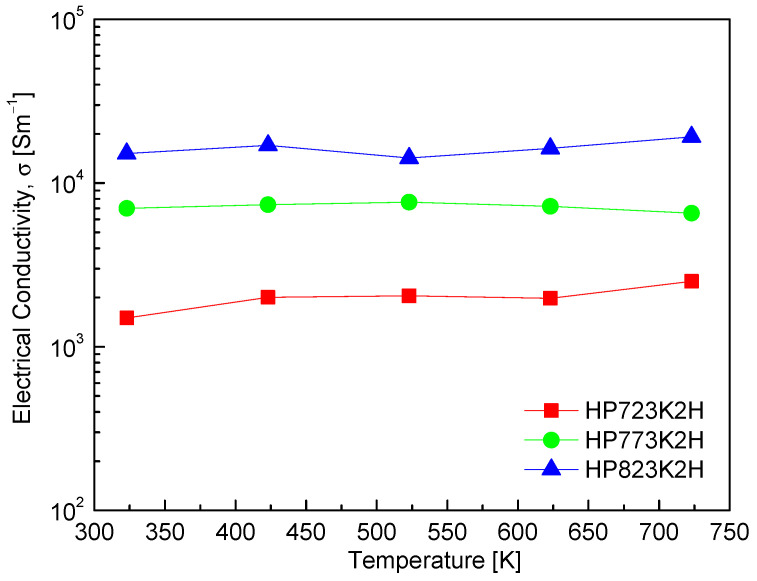
Electrical conductivity of CuFeS_2_ with different HP temperatures.

Figure 6 illustrates the electrical conductivity of CuFeS_2_ as a function of temperature and HP conditions. As the measurement temperature increased, a slight increase in electrical conductivity was observed. Notably, the electrical conductivity exhibited a significant increase with rising a HP temperature, escalating from 1.50 × 10^3^ to 1.51 × 10^4^ Sm^−1^ at 323 K and from 2.51 × 10^3^ to 1.92 × 10^4^ Sm^−1^ at 723 K. This pronounced enhancement in electrical conductivity is attributed to an increase in carrier (electron) concentration, which is likely due to sulfur deficiency arising from the elevated sintering temperatures. Li et al. [11] noted that in CuFeS_2_ produced by spark plasma sintering (SPS), sulfur vacancies contribute to an increase in carrier concentration, although they also lead to intensified carrier scattering, reducing mobility. As sintering temperatures rise, the loss of sulfur leads to the formation of vacancies, thus raising the number of available charge carriers and enhancing electrical conductivity. In their investigations, Li et al. [23] reported that the electrical conductivity of CuFeS_2_ exhibited a T^3/2^ dependence, indicative of dominant carrier scattering by ionized impurities within the temperature range of 300–700 K. Their findings highlight that ionized impurities create long-range Coulomb potential fields that scatter electrons, significantly diminishing mobility, which is a critical factor in conductivity. Conversely, Xie et al. [25] observed a negative temperature dependence of electrical conductivity in CuFeS_2_, attributing this phenomenon to a sharp decrease in electron mobility. They documented conductivity values in the range of (5.3–3.5) × 10^3^ Sm^−1^ between 300 and 630 K, illustrating variability in the reported electrical conductivity as a function of temperature and processing conditions. Further corroborating these observations, Ge et al. [11] provided an electrical conductivity range of (4.5–2.5) × 10^3^ Sm^−1^ at temperatures spanning 300–723 K. These variations in reported electrical conductivity across different studies highlight the complex interplay between synthesis methods, structural characteristics, and thermodynamic conditions influencing the electronic properties of CuFeS_2_.

**Figure 7:**
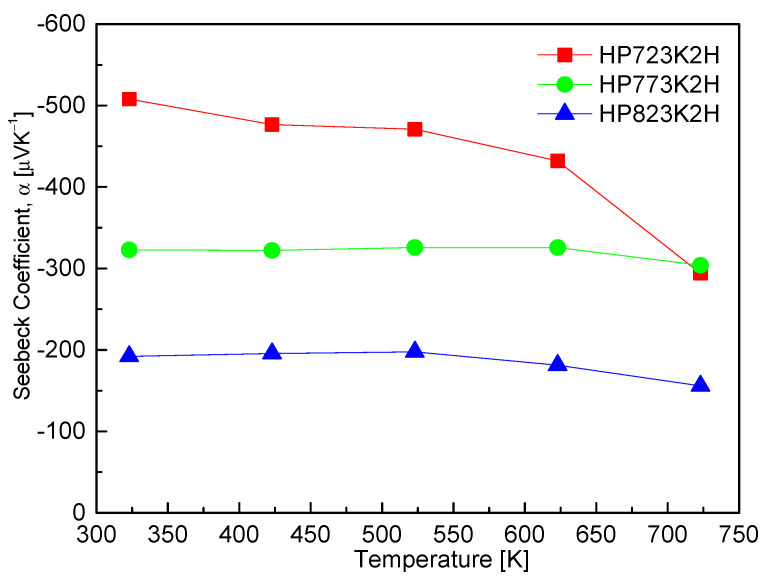
Seebeck coefficient of CuFeS_2_ with different HP temperatures.

Figure 7 shows the Seebeck coefficient of CuFeS_2_ across various samples, revealing that all measured values are negative. This negative sign indicates that chalcopyrite behaves as an *n*-type semiconductor. The Seebeck coefficient demonstrated a negative temperature dependence, which is typically inversely proportional to carrier concentration. Consequently, as the measurement temperature increased, the Seebeck coefficient exhibited a decreasing trend, contrasting with the behavior observed for electrical conductivity. In general, the Seebeck coefficient is expected to rise with increasing temperature, reaching a peak value before declining due to intrinsic transitions in the material’s electronic structure. Among the samples tested, the HP723K2H displayed the maximum Seebeck coefficient of −507.8 μVK^−1^ at 323 K. However, the HP823K2H showed a significant decline in the Seebeck coefficient across the entire temperature range. This sharp decrease can be attributed to the elevated carrier concentration resulting from sulfur deficiency and the presence of the talnakhite phase. Li et al. [11] reported a similar trend, indicating that the Seebeck coefficient of CuFeS_2_ diminished with increasing temperature during SPS. Specifically, when sintered at 873 K, the Seebeck coefficient decreased from −589 μVK^−1^ at 323 K to −449 μVK^−1^, and further decreased to below −400 μVK^−1^ at 573 K when sintered at 923 K. This observation supports the notion that higher processing temperatures can adversely affect the Seebeck coefficient due to the resulting changes in carrier dynamics. Furthermore, Carr et al. [9] noted a reduction in the Seebeck coefficient of CuFeS_2_ from a peak of −525 μVK^−1^ at 475 K to −365 μVK^−1^ at 670 K within the temperature range of 80–670 K. This indicates that while there is a tendency for the Seebeck coefficient to initially rise with temperature, it can decline sharply as the temperature increases beyond a certain threshold due to an increased carrier concentration. In contrast, Xie et al. [25] reported a minimum Seebeck coefficient of −362 μVK^−1^ at 470 K, after which it increased with temperature, reaching −380 μVK^−1^ at 630 K. This behavior suggests the possibility of complex carrier dynamics and phase transitions influencing the Seebeck coefficient in CuFeS_2_. Moreover, Ge et al. [16] observed an opposite temperature dependence, with the Seebeck coefficient peaking at −375 μVK^−1^ at 573 K, before declining to −300 μVK^−1^ at 723 K. This discrepancy emphasizes the intricate nature of the Seebeck coefficient’s response to temperature and processing conditions, indicating that factors such as the carrier concentration, material phase, and processing history play significant roles.

**Figure 8:**
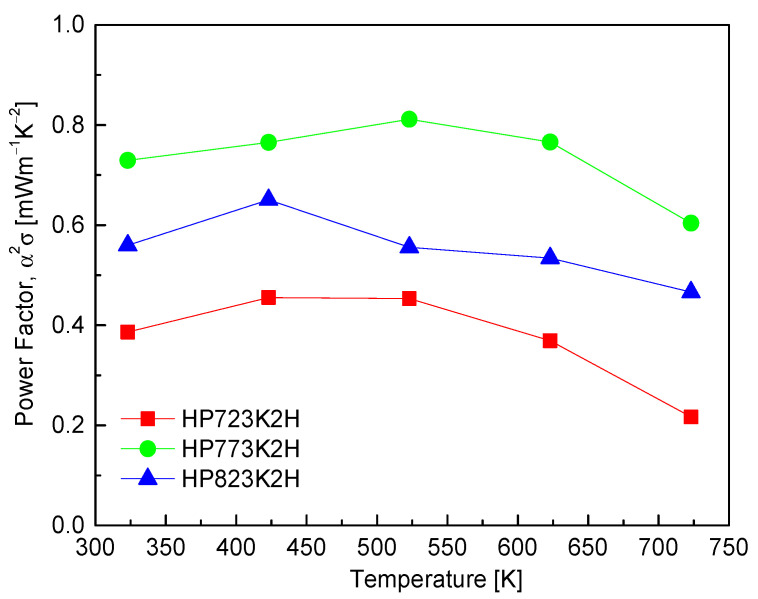
Power factor of CuFeS_2_ with different HP temperatures.

Figure 8 presents the PF values of CuFeS_2_, revealing that the PF reaches its maximum within the temperature range of 423–523 K. This peak can be attributed to the interplay between the temperature-dependent electrical conductivity and the Seebeck coefficient. Notably, the sample hot-pressed at 773 K (HP773K2H) exhibited consistently high PF values across the entire temperature range, culminating in a maximum PF of 0.81 mWm^−1^K^−2^ at 523 K. This performance significantly surpasses previously reported PF values for CuFeS_2_. For instance, Li et al. [11] reported a PF of 0.13 mWm^−1^K^−2^ at 573 K, while Carr et al. [9] found a value of 0.30 mWm^−1^K^−2^ at 570 K. Ge et al. [16] obtained a higher PF of 0.45 mWm^−1^K^−2^ at 523 K, yet this is still lower than the values observed in the current study. Moreover, Xie et al. [25] reported PF values of 0.69 mWm^−1^K^−2^ at 300 K and 0.51 mWm^−1^K^−2^ at 630 K, both of which are also lower than the maximum value achieved in this work.

The enhancement in the power factor observed in the HP773K2H sample is indicative of effective optimization in the thermoelectric properties of CuFeS_2_ through careful control of the HP conditions. The significant improvement in the PF can be linked to the favorable balance between high electrical conductivity and Seebeck coefficient, which is critical for achieving superior thermoelectric performance.

**Figure 9:**
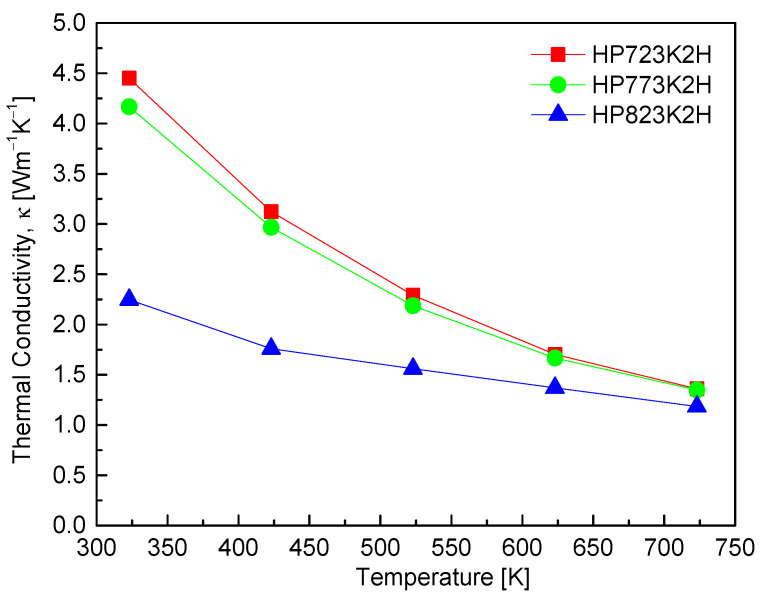
Thermal conductivity of CuFeS_2_ with different HP temperatures.

Figure 9 shows the thermal conductivity of CuFeS_2_, highlighting a notable trend: as both the measurement temperature and HP temperature increased, the thermal conductivity exhibited a decrease. The minimum thermal conductivity recorded was 1.36 Wm^−1^K^−1^ at 723 K for the HP823K2H sample. Despite the HP823K2H sample exhibiting the highest sintered density and electrical conductivity, its lower thermal conductivity can be attributed to the coexistence (composite) of the chalcopyrite and talnakhite phases, which likely enhances lattice scattering (phonon scattering). Thermal conductivity is a combination of the contributions from the phonon transports (lattice thermal conductivity) and the charge-carrier transports (electronic thermal conductivity), which can be separated using the Wiedemann–Franz law [26]. The HP823K2H sample displayed the lowest lattice thermal conductivity of 1.33 Wm^−1^K^−1^ at 723 K. The reduction in thermal conductivity with increasing temperature is a common phenomenon in thermoelectric materials, primarily due to enhanced phonon scattering mechanisms. Li et al. [23] noted a sharp decrease in thermal conductivity with rising measurement temperatures, attributing this behavior to strong Umklapp phonon scattering, which becomes more pronounced at elevated temperatures. This is consistent with the observations made by Carr et al. [9], who found that the thermal conductivity of CuFeS_2_ above room temperature followed a T^−1^ trend due to Umklapp scattering, with a minimum value of approximately 2 Wm^−1^K^−1^ at 670 K. Furthermore, Xie et al. [25] and Ge et al. [16] reported minimum thermal conductivities of 2.33 Wm^−1^K^−1^ at 630 K and 1.3 Wm^−1^K^−1^ at 723 K, respectively. Additionally, Li et al. [11] emphasized that as the SPS temperature rose, the sulfur content in CuFeS_2_ diminished, resulting in the introduction of more defects. These defects play a significant role in phonon scattering, thus lowering thermal conductivity. Their findings indicated minimum thermal conductivity values ranging from 5.5 to 2.5 Wm^−1^K^−1^ in the temperature range of 323–573 K for samples sintered at 923 K. These results corroborate the trends observed in this study, reinforcing the idea that increased temperatures and defect concentrations contribute significantly to phonon scattering mechanisms in CuFeS_2_.

**Figure 10:**
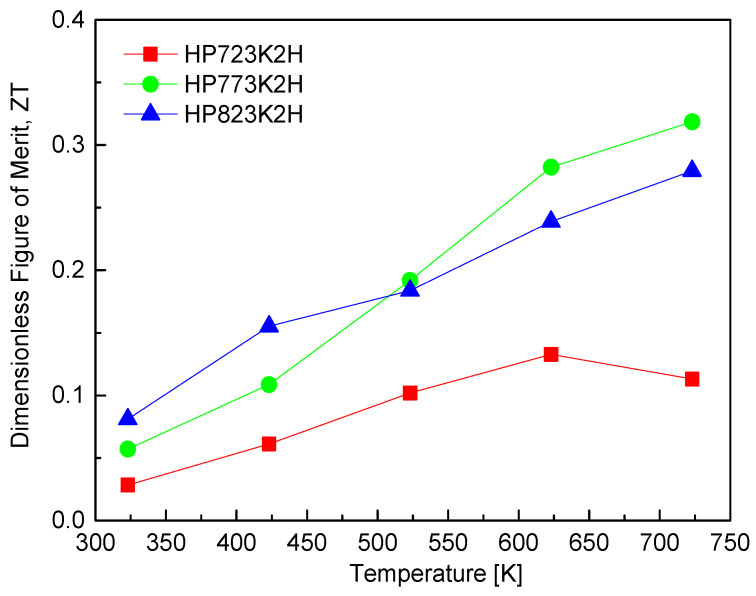
ZT values of CuFeS_2_ with different HP temperatures.

Figure 10 illustrates the ZT values of CuFeS_2_, exhibiting a significant enhancement in thermoelectric performance as the temperature increased. Initially, at 323 K, the ZT values were relatively low, ranging from 0.01 to 0.09. However, due to the optimized HP conditions and the increase in measurement temperature, the HP773K2H sample achieved a notable improvement in ZT, reaching 0.32 at 723 K. The ZT value is a critical parameter for assessing the efficiency of thermoelectric materials, where higher values indicate better thermoelectric performance. The results from this study align well with previous findings in the literature. Li et al. [11] reported ZT values for CuFeS_2_ produced via mechanical alloying followed by spark plasma sintering (MA–SPS) that ranged from 0.03 to 0.07 at 573 K, varying with the milling time and SPS temperature. Similarly, Li et al. [23] achieved a ZT of 0.22 at 700 K for CuFeS_2_ synthesized through a melting-annealing–SPS method, highlighting the influence of synthesis techniques on thermoelectric performance. In a comparative context, Carr et al. [9] reported a ZT of 0.09 at 670 K for CuFeS_2_ produced through a combination of melting, annealing, quenching, and high-energy ball milling, followed by HP. Xie et al. [5] obtained a ZT of 0.13 at 630 K using a melting process combined with plasma activated sintering (PAS), while Ge et al. [16] achieved a ZT of 0.15 at 723 K with a similar PAS approach. Tippireddy et al. [27] employed a multi-step process—vacuum melting, crushing, remelting, crushing, and HP—to produce Cr-doped chalcopyrite; For Cu_0.92_Cr_0.08_FeS_2_, a ZT of 0.31 was achieved at 673 K; however, undoped CuFeS_2_ exhibited a ZT of 0.08 at 673 K, which is significantly lower than our ZT of 0.32 at 723 K. In this study, we synthesized a single-phase undoped chalcopyrite through a simple MA–HP process, achieving a thermoelectric performance comparable to Tippireddy et al.’s Cr-doped samples. The findings indicate that the MA–HP method employed in this study not only allows for the successful synthesis of chalcopyrite CuFeS_2_ but also provides a pathway to achieve higher thermoelectric performance. The enhanced ZT value of 0.32 at 723 K demonstrates the effectiveness of controlling the HP temperature and the resulting microstructural properties, which contribute significantly to the thermoelectric performance of CuFeS_2_.

## 4. Conclusions

The controlled mechanical alloying time and hot pressing temperature facilitated the successful synthesis of tetragonal chalcopyrite CuFeS_2_ through solid-state processing. A comprehensive thermal analysis, including thermogravimetric and differential scanning calorimetry, in conjunction with an X-ray diffraction phase analysis, led to the determination of optimal MA conditions at 350 rpm for 12 h. The HP conditions were subsequently optimized to 773 K for 2 h, which effectively preserved the chalcopyrite phase without secondary phase formation. In contrast, HP at 823 K resulted in the formation of a secondary phase, talnakhite (Cu_1.1_Fe_1.1_S_2_), attributed to sulfur volatilization. This phenomenon underscores the importance of controlling processing temperatures to maintain the integrity of the desired chalcopyrite phase. The synthesized chalcopyrite CuFeS_2_ exhibited *n*-type transport characteristics throughout the measured temperature range, indicating its potential utility as a thermoelectric material. Notably, an increase in the HP temperature correlated with a rise in electrical conductivity, while both the Seebeck coefficient and thermal conductivity exhibited a decreasing trend. This behavior is believed to be linked to sulfur volatilization and the consequent formation of the talnakhite phase, which likely enhanced carrier concentration while reducing mobility. Among the samples tested, the specimen processed under the HP condition of 773 K (HP773K2H) demonstrated the highest power factor of 0.81 mWm^−1^K^−2^ at 523 K, culminating in a maximum dimensionless figure-of-merit of 0.32 at 723 K. In conclusion, the tetragonal chalcopyrite CuFeS_2_, exhibiting semiconductor behavior, can be efficiently synthesized via the MA–HP method. This research highlights its promising application as an *n*-type thermoelectric material, paving the way for further exploration and the optimization of its thermoelectric properties in practical applications.

## Figures and Tables

**Table 1:** Specimen designations and processing variables for chalcopyrite CuFeS_2_ prepared via MA–HP process.

Specimen	Mechanical Alloying	Hot Pressing
MA350R6H	at 350 rpm for 6 h	-
MA350R12H	at 350 rpm for 12 h	-
MA350R18H	at 350 rpm for 18 h	-
MA350R24H	at 350 rpm for 24 h	-
HP723K2H	at 350 rpm for 12 h	at 723 K for 2 h under 70 MPa
HP773K2H	at 350 rpm for 12 h	at 773 K for 2 h under 70 MPa
HP823K2H	at 350 rpm for 12 h	at 823 K for 2 h under 70 MPa

**Table 2:** Relative density, crystallite size, and lattice parameters of tetragonal chalcopyrite CuFeS_2_ prepared via MA–HP process.

Specimen	Relative Density [%]	Crystallite Size [nm]	Lattice Parameter		
			a [nm]	c [nm]	c/a
MA350R6H	-	14.5	0.5283	1.0375	1.9638
MA350R12H	-	14.0	0.5280	1.0364	1.9630
MA350R18H	-	13.5	0.5282	1.0365	1.9623
MA350R24H	-	12.8	0.5288	1.0380	1.9630
HP723K2H	96.9	36.9	0.5289	1.0419	1.9699
HP773K2H	98.9	112.6	0.5292	1.0438	1.9724
HP823K2H	99.2	135.8	0.5289	1.0418	1.9697

## Data Availability

The original contributions presented in this study are included in the article; further inquiries can be directed to the corresponding author.
